# From Psychiatry to Neurology and Endocrinology: A Case of Hypoparathyroidism

**DOI:** 10.7759/cureus.10666

**Published:** 2020-09-26

**Authors:** Saurabh Gaba, Mandeep Singla, Monica Gupta, Ashish Dua, Nayana Gaba

**Affiliations:** 1 General Medicine, Government Medical College and Hospital, Chandigarh, IND; 2 Radiodiagnosis, Government Medical College and Hospital, Chandigarh, IND; 3 Obstetrics and Gynaecology, Postgraduate Institute of Medical Education and Research, Chandigarh, IND; 4 Obstetrics and Gynaecology, Government Medical College and Hospital, Chandigarh, IND

**Keywords:** hypoparathyroidism, hypocalcemia, depression, basal ganglia, calcification, parkinsonism, trousseau, chvostek, qt interval, extrapyramidal

## Abstract

A 31-year-old male patient developed extrapyramidal symptoms while on treatment for depression. He was investigated and found to suffer from hypoparathyroidism. He had calcification in the brain, signs and symptoms of neuromuscular irritability, and QT prolongation on electrocardiogram. He was treated with calcium carbonate and calcitriol. Although he had marked improvement, bradykinesia persisted. This report highlights the importance of maintaining a high index of suspicion for hypocalcemia, and the importance of searching for an organic basis for psychiatric symptoms.

## Introduction

Hypoparathyroidism can present overtly or covertly with a wide range of clinical manifestations [1]. Although the diagnosis can be made easily by blood investigations, it can sometimes be delayed due to the non-specific nature of the symptoms and an unsuspecting physician. This report describes one such case where a patient was being treated for depression and was later investigated after he developed extrapyramidal symptoms. This led to the revelation of a vivid biochemical profile of hypoparathyroidism with hypocalcemia, hyperphosphatemia, and low serum intact parathyroid hormone (PTH), and extensive calcification in the brain.

## Case presentation

**Figure 1:**
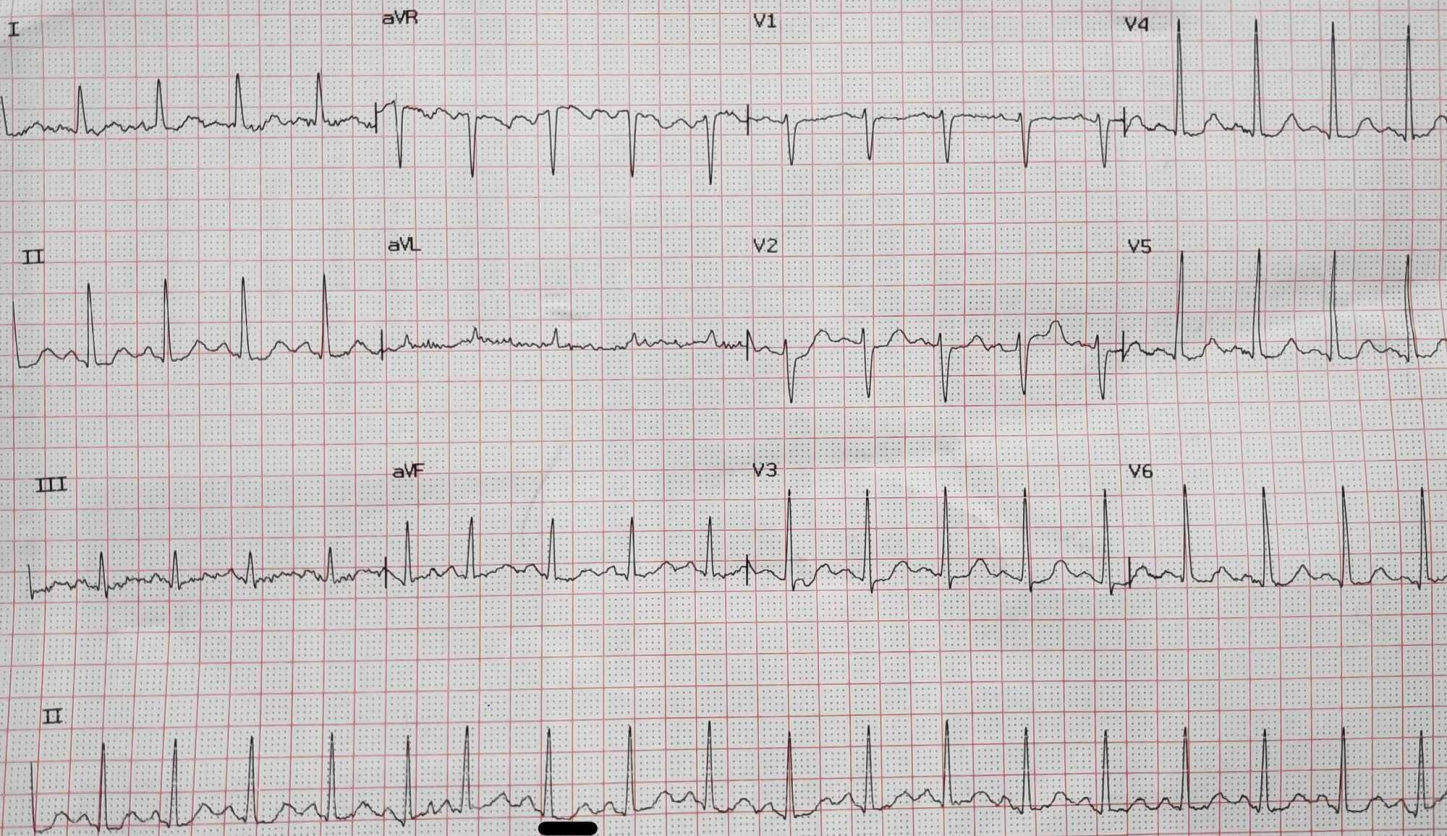
ECG showing sinus tachycardia with QTc prolongation (451 ms) ECG, electrocardiogram; QTc, corrected QT The QT interval is marked with a black bar at the bottom of the figure.

**Figure 2:**
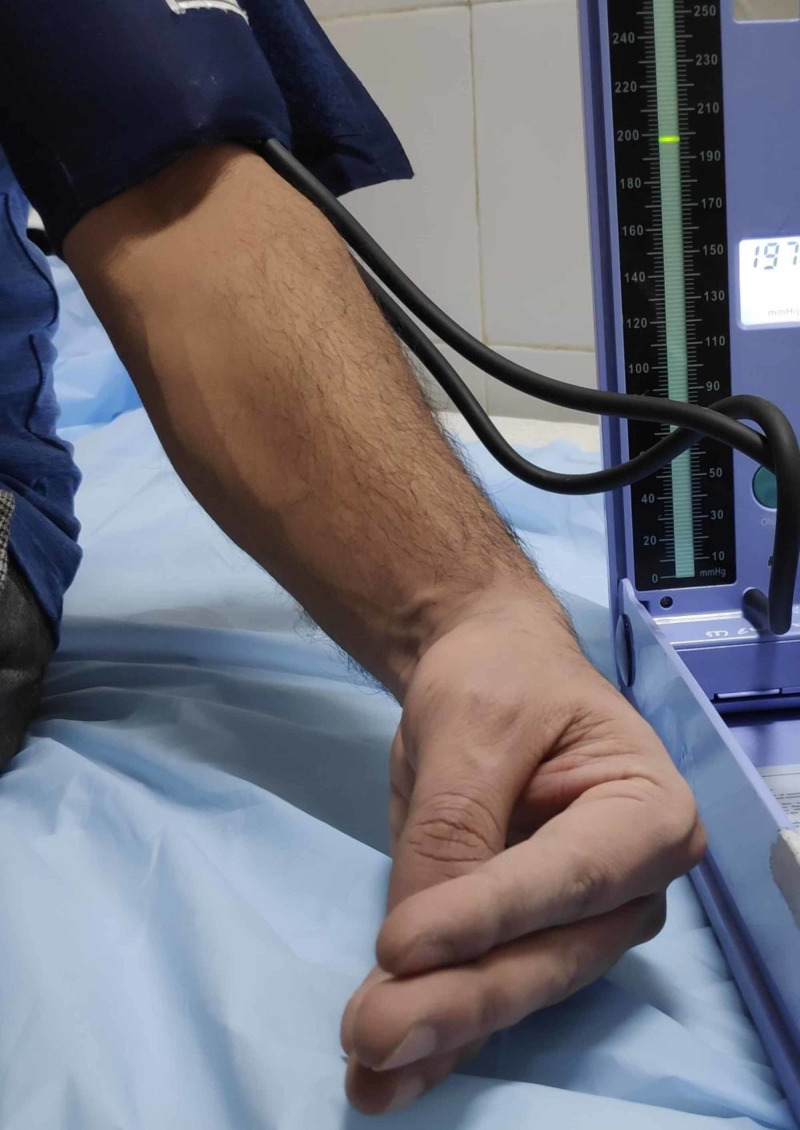
Trousseau’s sign Carpal spasm occurring after the cuff had been inflated to a pressure above the systolic blood pressure.

**Figure 3:**
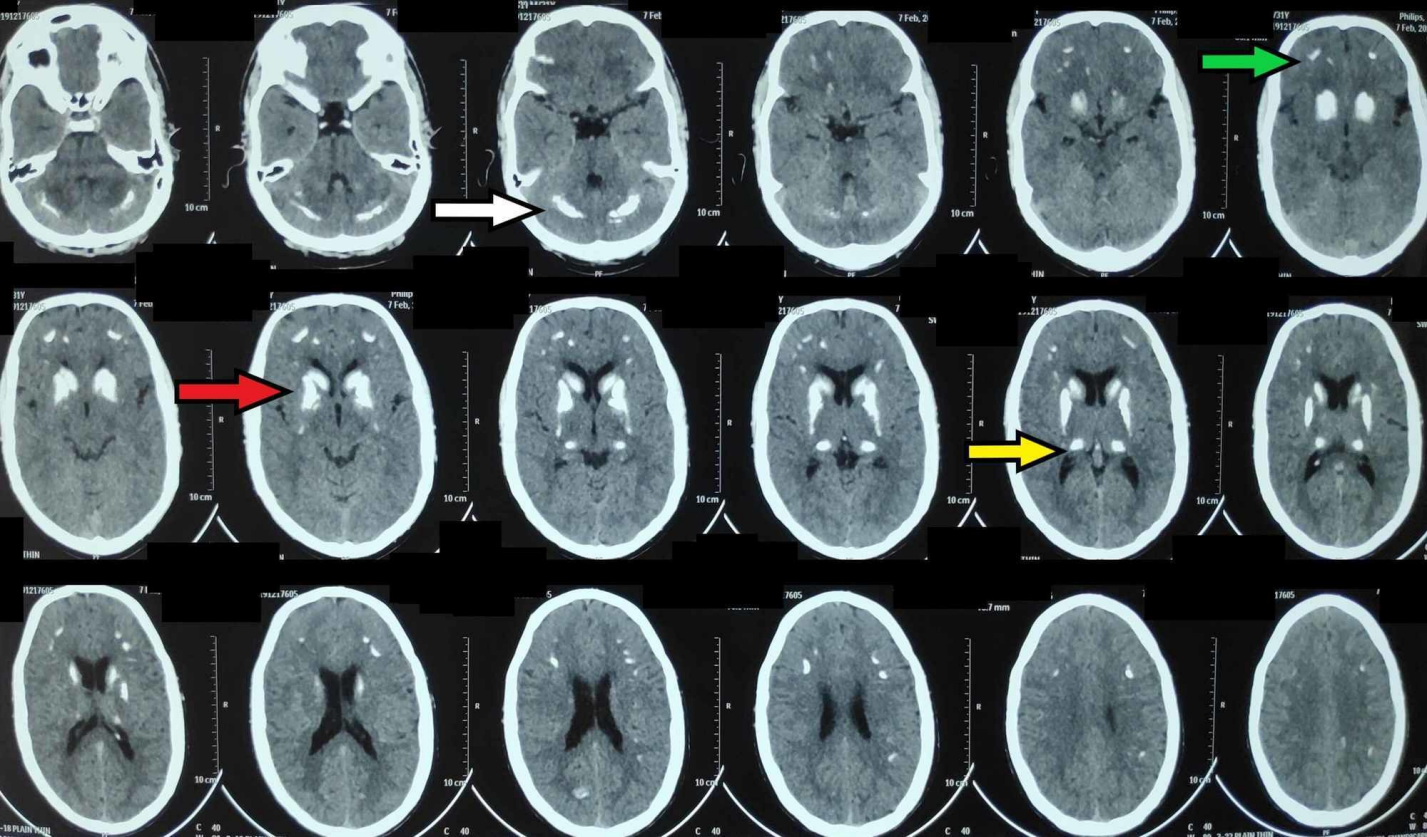
Axial sections of a CT scan of the brain showing bilateral symmetrical calcification CT, computed tomography The calcification is evident as hyperdensity, predominantly in bilateral basal ganglia (red arrow) and thalami (yellow arrow). Smaller lesions are also seen in the frontal cortex (green arrow) and cerebellum (white arrow).

**Figure 4:**
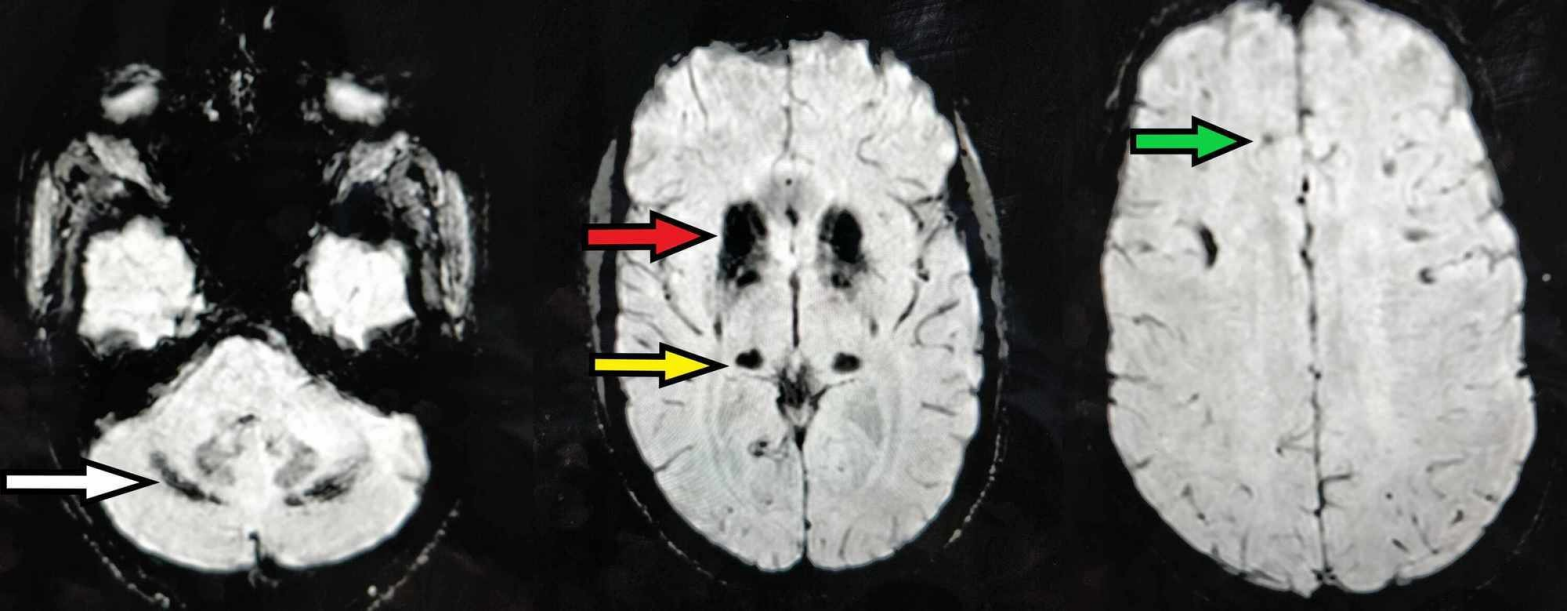
Axial sections of the SWI sequence of MRI brain showing bilateral symmetrical calcification SWI, susceptibility-weighted imaging; MRI, magnetic resonance imaging The calcification is evident as blooming, predominantly in bilateral basal ganglia (red arrow) and thalami (yellow arrow). Smaller lesions are also seen in the frontal cortex (green arrow) and cerebellum (white arrow).

**Figure 5:**
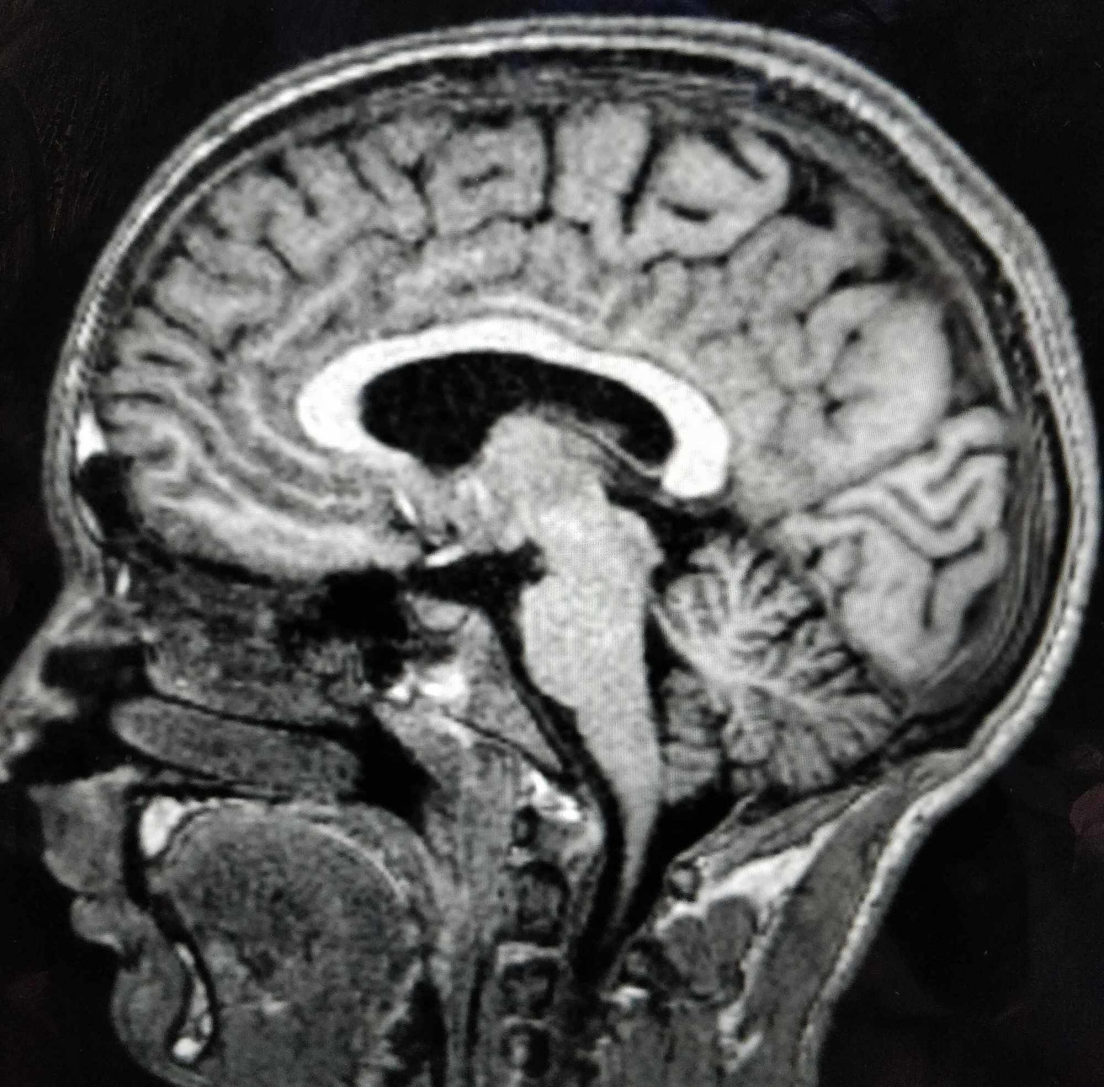
Saggital section of the T1W sequence of MRI brain T1W, T1-weighted; MRI, magnetic resonance imaging No structural abnormality is seen in the brainstem.

A 31-year-old man presented to the internal medicine outpatient department after being referred by a psychiatrist who was managing him for depression with amitriptyline. He was on treatment for over a year and the current referral was prompted by the finding of generalized slowing of body movements. He was fidgety and his gait, albeit slow, was not otherwise peculiar. He reported symptoms of low mood, fatigue, aches, poor sleep, vertigo, and numbness over the face and limbs. He had easily discernible bradykinesia, which he corroborated by giving a history of requiring more time, as compared to what he required a few months back, to perform any activity such as bathing, dressing, cooking, and eating. This had adversely impacted his work as a domestic helper. His limbs were mildly hypertonic, deep tendon reflexes were normal, and there were no tremors. His cranial nerve, sensory, and cerebellar examinations were normal. The mini-mental state examination (MMSE) revealed mild cognitive impairment. No other abnormality was found on systemic examination. He had an average built without any dysmorphic physical features. The initial panel of investigations revealed hypocalcemia and hyperphosphatemia, for which another set of investigations was done that led to a diagnosis of isolated hypoparathyroidism (Table 1). There was no history of neck surgery. An electrocardiogram (ECG) showed a prolonged corrected QT interval, and Trousseau’s sign was elicitable (Figures 1-2). A computed tomography (CT) scan of the brain revealed extensive calcification (Figure 3). A magnetic resonance imaging (MRI) scan corroborated the findings seen on the CT scan (Figure 4) and ruled out other structural pathology that could have led to idiopathic Parkinson's disease and Parkinson-plus syndromes (Figure 5). He was treated with calcium carbonate and calcitriol, and amitriptyline was discontinued. With the control of metabolic parameters, his general well-being and mood improved, but the extrapyramidal symptoms persisted.

**Table 1:** Clinical investigations iPTH, intact parathyroid hormone; TSH, thyroid-stimulating hormone; T3, triiodothyronine; T4, tetraiodothyronine *Corrected calcium = measured total calcium + 0.8 (4.0 - serum albumin)

Investigation	Value	Normal range
Hemoglobin (g/dL)	13.2	13-16
Total leukocyte count (X10^9/L)	6.3	4-12
Platelets (X10^9/L)	192	150-400
Sodium (mmol/L)	142	135-145
Potassium (mmol/L)	4.2	3.5-5.5
Urea (mg/dL)	30	15-40
Creatinine (mg/dL)	1	<1.3
Corrected calcium* (mg/dL)	6.9	8-10.4
Phosphorus (mg/dL)	7	2.5-4.5
Magnesium (mg/dL)	2.1	1.2-2.5
Aspartate transaminase (U/L)	32	10-40
Alanine transaminase (U/L)	28	10-40
Total protein (gm/dL)	7	6-8
Albumin (gm/dL)	3.9	3.5-5.5
25-hydroxy vitamin D (ng/mL)	27	30-100
iPTH (pg/mL)	10.75	12-88
24-hour urinary calcium (mg)	74	100-300
TSH (mIU/L)	3.2	0.5-5
Free T3 (pg/dL)	270	260-480
Free T4 (ng/dL)	1.2	0.7-1.8

## Discussion

PTH is the major hormone that regulates the serum calcium level. It increases the serum calcium by mobilizing it from the bones, increasing renal tubular resorption, and increasing the intestinal absorption [2]. High serum ionized calcium provides a negative feedback signal to the parathyroid glands and inhibits the secretion of PTH. The causes of hypoparathyroidism are mentioned in Table 2 [3].

**Table 2:** Causes of hypoparathyroidism

Causes of hypoparathyroidism
Neck surgery (most common)
Genetic defects
Isolated autoimmune hypoparathyroidism
Autoimmune polyglandular syndrome type 1
Inherited syndromes – DiGeorge syndrome, Kearns-Sayre syndrome, mitochondrial encephalopathy with lactic acidosis and stroke-like episodes (MELAS) syndrome
Hemochromatosis
Irradiation
Metastasis

The biochemical hallmarks of hypoparathyroidism are hypocalcemia and hyperphosphatemia in the setting of a low or inappropriately normal level of intact PTH in the serum [4]. If the unionized serum calcium level is measured, it should be corrected for albumin. The serum vitamin D level is not affected in hypoparathyroidism. Magnesium deficiency should always be ruled out, as it can induce reversible PTH resistance, producing the clinical manifestations of hypoparathyroidism. The clinical features depend on the severity and duration of hypocalcemia. These are summarized in Table 3 [1,5].

**Table 3:** Clinical features of hypoparathyroidism Trousseau’s sign refers to the carpal spasm produced by the inflation of the sphygmomanometer cuff 20 mmHg above the systolic blood pressure for three minutes. Chvostek’s sign is the twitching of facial muscles on tapping the facial nerve anterior to the ear. Extrapyramidal symptoms are resting tremors, rigidity, bradykinesia, and postural instability.

System	Features
Neuromuscular	Perioral numbness, paresthesias, cramps, tetany, laryngospasm, carpopedal spasm, Trousseau’s sign, Chvostek’s sign
Cardiovascular	QT interval prolongation on electrocardiogram, polymorphic ventricular tachycardia (torsade de pointes), hypotension, heart failure
Neuropsychiatric	Anxiety, irritability, depression, psychosis, seizures, papilledema, basal ganglia calcification, dementia, extrapyramidal symptoms
Others	Dry skin, alopecia, brittle nails, dental hypoplasia, defective tooth enamel, cataract, osteoporosis

Basal ganglia calcification, especially in the globus pallidus, can occur physiologically as part of ageing. The other causes of basal ganglia calcification are mentioned in Table 4 [6].

**Table 4:** Causes of basal ganglia calcification CNS, central nervous system; TORCH, toxoplasmosis, others (syphilis, varicella-zoster, parvovirus B19), rubella, cytomegalovirus, and herpes

Causes of basal ganglia calcification
Idiopathic (Fahr disease)
Hypoparathyroidism
Pseudohypoparathyroidism
Hyperparathyroidism
Tuberous sclerosis
Carbon monoxide poisoning
Lead poisoning
Neurocysticercosis
CNS tuberculosis
TORCH infections

The treatment of hypoparathyroidism involves maintaining the serum calcium in the lower half of the normal range (8-9 mg/dL) by calcium and vitamin D supplementation. Calcitriol (1,25-dihydroxy vitamin D) is more effective than alphacalcidol (1-hydroxy vitamin D) [7]. Usually, 1000-2000 mg of elemental calcium and 0.25-2 mcg of calcitriol per day are required. An important aspect of management is the periodic measurement of 24-hour urinary calcium excretion [8]. In the absence of PTH, the renal tubular resorption of calcium is adversely affected, leading to hypercalciuria. This can lead to renal stones, nephrocalcinosis, and chronic kidney disease. The urine calcium excretion should be kept below 300 mg in 24 hours by adjusting the dose of calcium and vitamin D. A thiazide diuretic may also be added, as it decreases urinary calcium loss [9]. For severe hypocalcemia associated with cardiovascular and neurological dysfunction or for the symptoms of neuromuscular irritability occurring acutely after hypoparathyroidism due to neck surgeries such as total or near-total thyroidectomy, intravenous infusion of 10% calcium gluconate added to 5% dextrose is given [10]. Postsurgical hypoparathyroidism can be transient and it may resolve after a few months.

The other important clinical differential for a patient presenting with psychiatric and extrapyramidal symptoms, as in the case under consideration in this report, is Wilson’s disease; however, it is also frequently accompanied by dysarthria, ataxia, and movement disorders such tremors and chorea [11]. Psychosis and dementia can also occur. Liver involvement is a cardinal feature of Wilson’s disease, and it can vary from asymptomatic transaminitis to decompensated cirrhosis. The unequivocal laboratory and radiological evidence of hypoparathyroidism in our case, along with the absence of any movement disorder and hepatic abnormality, did not justify further workup for Wilson’s disease. Similarly, there was no circumstantial evidence to consider the other causes of Parkinsonism such as encephalitis, stroke, carbon monoxide poisoning, and drug-induced Parkinsonism [12].

## Conclusions

This report describes a 31-year-old male who was being treated for depression and subsequently diagnosed to be suffering from hypoparathyroidism after developing extrapyramidal symptoms attributable to basal ganglia calcification. He was also found to have mild cognitive impairment, QT interval prolongation on ECG, and Trousseau’s sign. He was treated with calcium carbonate and calcitriol, which improved his mood and neuromuscular irritability symptoms, but the extrapyramidal features persisted. This highlights the importance of investigating patients with suspected primary psychiatric disorders to rule out organic pathologies.
